# Anatomical Consideration for Double Chin Thread Lifting

**DOI:** 10.1111/jocd.70238

**Published:** 2025-06-13

**Authors:** Gi‐Woong Hong, Jovian Wan, Song‐Eun Yoon, Sky Wong, Kyu‐Ho Yi

**Affiliations:** ^1^ Samskin Plastic Surgery Clinic Seoul Korea; ^2^ Medical Research Inc. Wonju Korea; ^3^ Brandnew Aesthetic Surgery Clinic Seoul Korea; ^4^ LEciel Medical Centre Hong Kong; ^5^ Human Identification Research Institute, BK21 FOUR Project Division in Anatomy and Developmental Biology, Department of Oral Biology Yonsei University College of Dentistry Seoul Korea; ^6^ You and I Clinic Seoul Korea

**Keywords:** cervical fascia, mentum, minimally invasive surgical procedures, platysma muscle, rhytidoplasty

## Abstract

**Background:**

Thread lifting is a popular minimally invasive option for correcting submental fullness. Given the complex anatomy and multifactorial causes of double chin deformity, anatomical understanding is critical for safe and effective treatment. This study outlines key anatomical considerations and procedural strategies for optimal outcomes in double chin thread lifting.

**Methods:**

This review integrates cadaveric dissection, ultrasound imaging, and clinical experience to analyze cervical anatomy, focusing on platysmal patterns, fascial layers, and neurovascular landmarks. Two thread techniques (I‐shaped and U‐shaped cogged threads) are compared, with emphasis on safer fixation using the subangular deep fascia.

**Results:**

Korean patients exhibit a high prevalence of platysmal decussation (85%). Anchoring to the subangular deep fascia avoids critical structures such as the great auricular nerve and parotid gland, offering enhanced safety over traditional mastoid fixation. Medial‐to‐lateral thread insertion with reverse‐specific cogs improves central submental traction. Ultrasound guidance further enhances procedural accuracy.

**Conclusion:**

Using the subangular deep fascia as an anchor point improves safety and effectiveness in double chin thread lifting. Individualized planning based on anatomical variation, fat volume, and platysmal type is essential for optimal and lasting results.

## 1 Introduction

The correction of submental fullness, commonly known as double chin deformity, represents a significant challenge in aesthetic medicine due to its multifactorial etiology and complex anatomical considerations. Understanding the interplay between skeletal structure, soft tissue layers, and muscular anatomy is crucial for successful intervention [1, 2, 3, 4, 5, 6].

The cervical region's layered structure, comprising skin, subcutaneous fat, platysma muscle, subplatysmal fat, cervical fascia, and deep muscles, creates a complex environment for thread‐based interventions. The variation in platysmal decussation patterns, particularly between Asian and Western populations, significantly influences treatment approaches and outcomes [7, 8, 9, 10].

Recent advances in thread design and placement techniques have expanded the possibilities for non‐surgical double chin correction. However, successful outcomes require careful consideration of neurovascular structures, salivary glands, and supporting fascia [11, 12].

The evolution of anchor point selection, moving from traditional mastoid fascia fixation to the utilization of subangular deep fascia, represents a significant advancement in technique safety and efficacy, particularly in avoiding critical anatomical structures [13].

## 2 Anatomical Considerations

**FIGURE 1:**
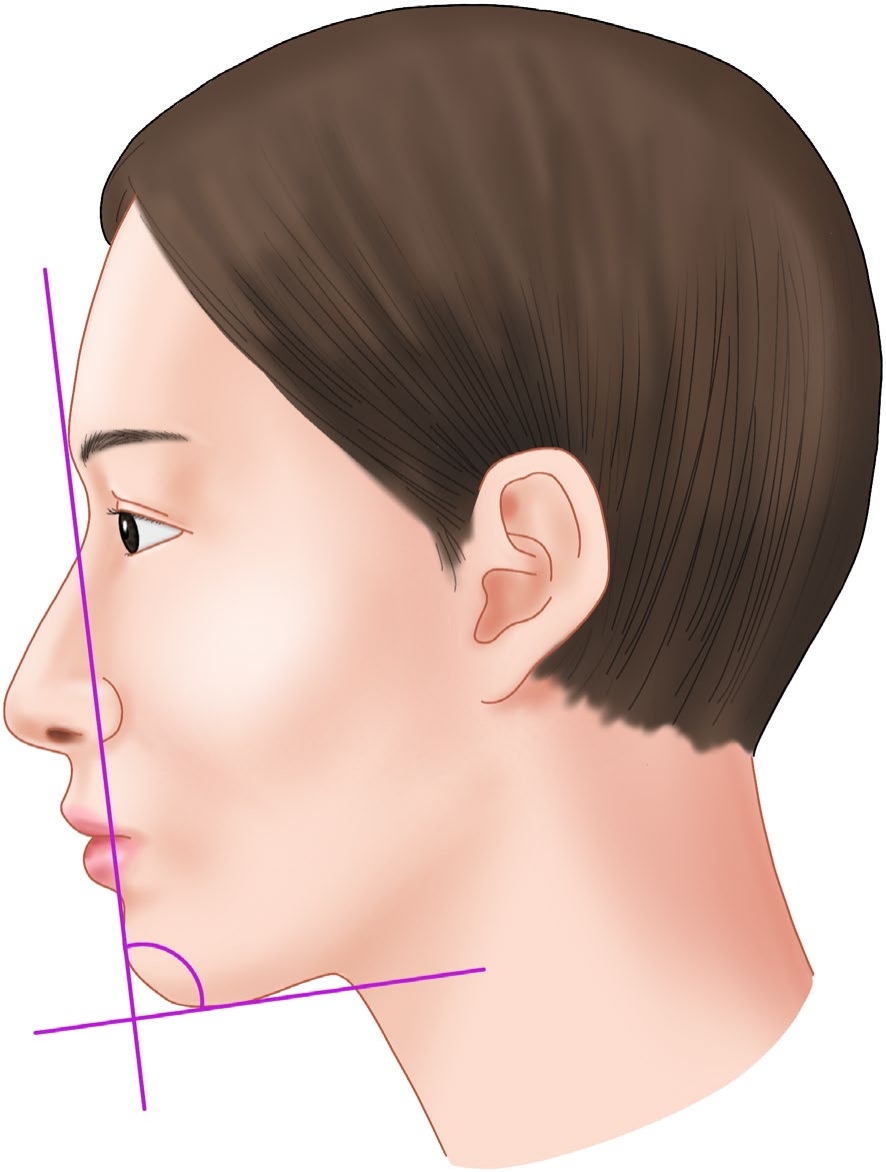
Anatomical illustration demonstrating the mentocervical angle formation, where a line drawn from the cervical point to the menton intersects with the anterior facial plane. The ideal range for this angle is between 80° to 95°, with deviation indicating submental fullness.

Double chin deformity, typically characterized by submental fullness, arises from multiple etiologies. This condition results in an increase in the mentocervical angle—the angle formed by the intersection of the line from the cervical point to the menton and the anterior facial plane. Normally ranging from 80° to 95°, this angle becomes more obtuse with the progression of submental protrusion (Figure 1) [14, 15].

To comprehend double chin deformity, a thorough understanding of cervical anatomy is essential. The cervical region is bounded superiorly by the mandibular lower border and mastoid process, inferiorly by the clavicle, and posteriorly by the trapezius muscle. The neck is conventionally divided into six layers, from superficial to deep: skin, subcutaneous fat, platysma muscle, subplatysmal fat, cervical fascia, and deep muscles with the submandibular gland [16, 17, 18].

**FIGURE 2:**
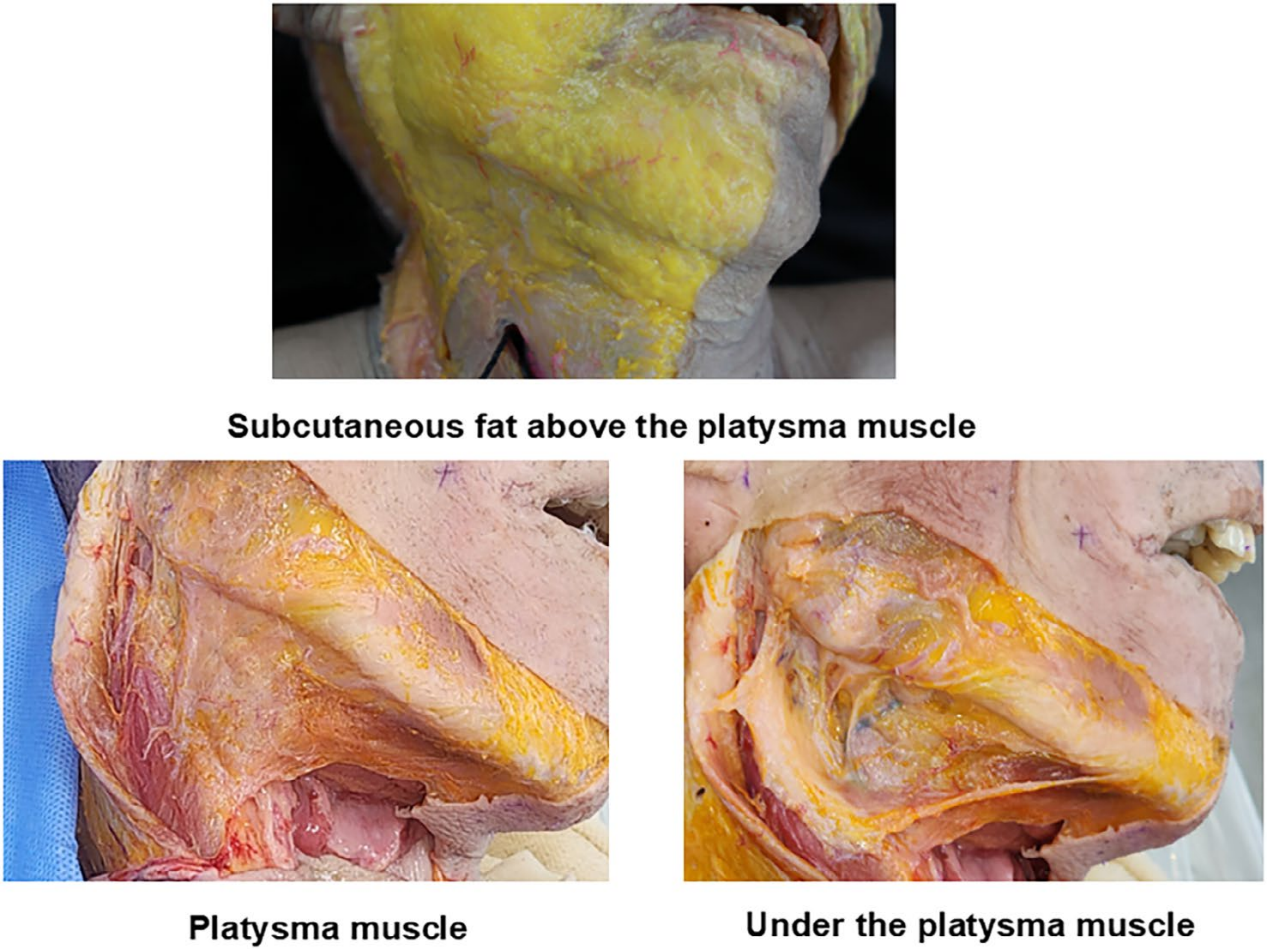
Cross‐sectional diagram showing the six distinct anatomical layers of the neck, from superficial to deep: Skin, subcutaneous fat, platysma muscle, subplatysmal fat, cervical fascia, and deep muscles with submandibular gland.

The platysma muscle originates from the fasciae of the pectoralis major and deltoid muscles in the upper chest, ascends over the clavicle, and extends to the lower face, enveloping the anterior neck. While a portion of its anterior fibers attaches to the mandibular border, the majority of its lateral fibers continue superiorly, deep to the depressor anguli oris and risorius muscles, merging medially with the modiolus. Laterally, it overlies the masseter muscle and parotid gland, forming a continuum with the midfacial SMAS (Figure 2) [19].

**FIGURE 3:**
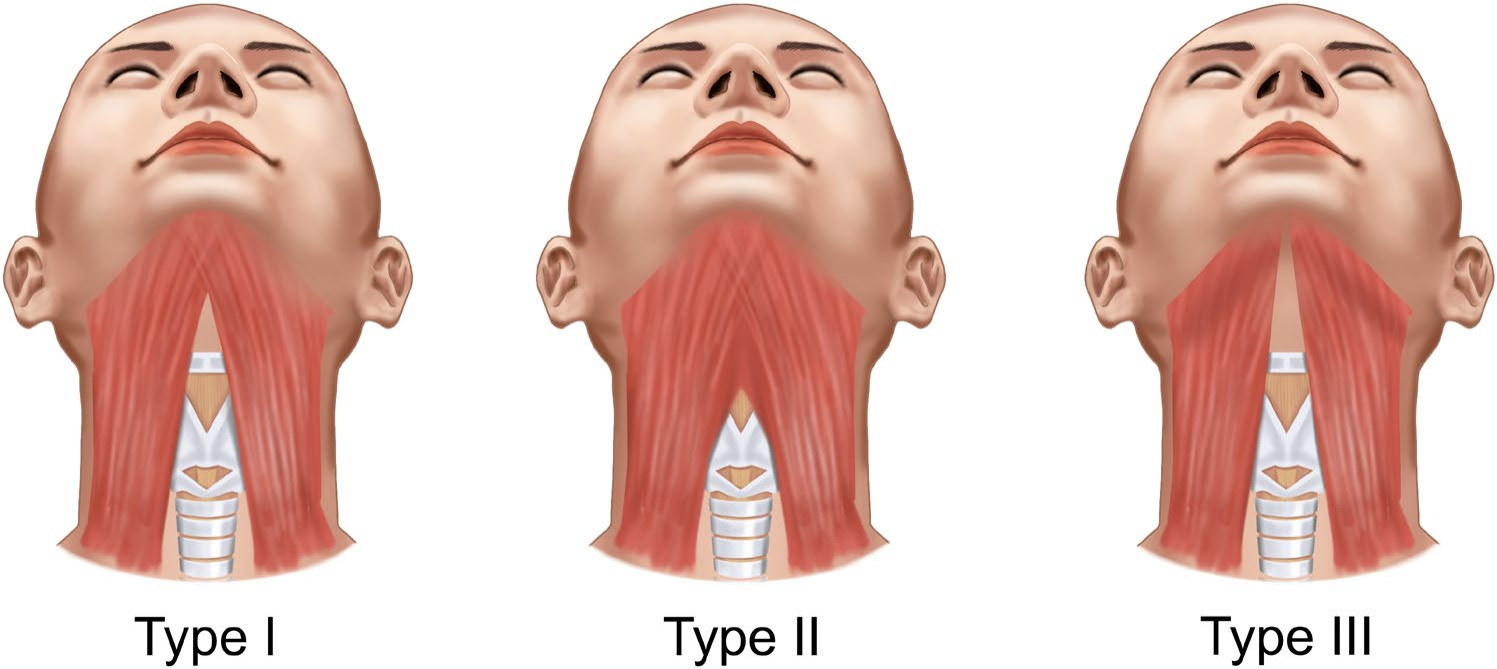
Detailed anatomical illustration depicting muscle attachment patterns to the internal mandibular surface, emphasizing the role of muscular support in submental contour.

Inferiorly, the platysma muscle bifurcates, flanking the cervical cartilages, and converges superiorly. The level of this bifurcation point varies among individuals, influencing the degree of support provided to the underlying structures. While the mid‐cervical region typically lacks platysmal fibers, the submental region often exhibits decussation of the left and right muscle bellies. This decussation pattern is classified into three types: Type I (< 2 cm width of decussation), Type II (≥ 2 cm width of decussation), and Type III (no decussation) (Figure 3) [20].

Recent statistics indicate that in the Korean population, over 85% exhibit platysmal decussation, with Type I accounting for 40%, Type II for 45%, and Type III for 15%. In contrast, Western populations show a higher prevalence of Type I (> 50%). 75% of South Americans demonstrated Type I pattern, 15% for type II, and 10% for type III [21]. Type I was the majority in Caucasians (61%), followed by Type II (39%). Additionally, non‐Asian patients often exhibit thicker platysmal muscles with deeper cervical fascial attachments, potentially requiring stronger thread designs and modified insertion techniques. Practitioners should adjust their approach based not only on individual assessment but also considering these population‐based anatomical tendencies [22]. The absence of platysmal decussation predisposes individuals to more severe double chin deformities, colloquially termed “turkey neck” or “gobbler neck deformity.” In the elderly, this can manifest as significant skin laxity and fat accumulation along the medial and lateral borders of the muscle [20].

Severe turkey neck deformities in the elderly often necessitate surgical interventions such as corset platysmaplasty. Fortunately, the Korean population exhibits a lower incidence of Type III platysma and generally less developed cervical musculature compared to Western counterparts, resulting in a lower prevalence of severe neck deformities requiring surgical correction. Consequently, Korean patients more commonly present with horizontal neck rhytids, skin laxity due to loss of elasticity, double chin deformity, and platysmal banding associated with subcutaneous fat atrophy. These issues are particularly prevalent in males, lean individuals, and those with increased cervical mobility [13, 23, 24].

Even in cases without Type III platysma, a wide muscle bifurcation can facilitate the protrusion of deep fat layers, resulting in double chin deformity. The etiology of double chin deformity can be broadly categorized into three main causes:

1. Congenital factors.
2. Latrogenic causes, such as two‐jaw surgery or genioplasty.
3. Excessive submental fat accumulation.

Congenital cases often present with mandibular hypoplasia accompanied by a “turkey neck” appearance. Insufficient anterior projection of the chin leads to a reduced anterior–posterior length of the mandible, from the most prominent point of the chin to the cervical point. This shortening can result in a concentration of soft tissues, including fat, contributing to submental fullness.

**FIGURE 4:**
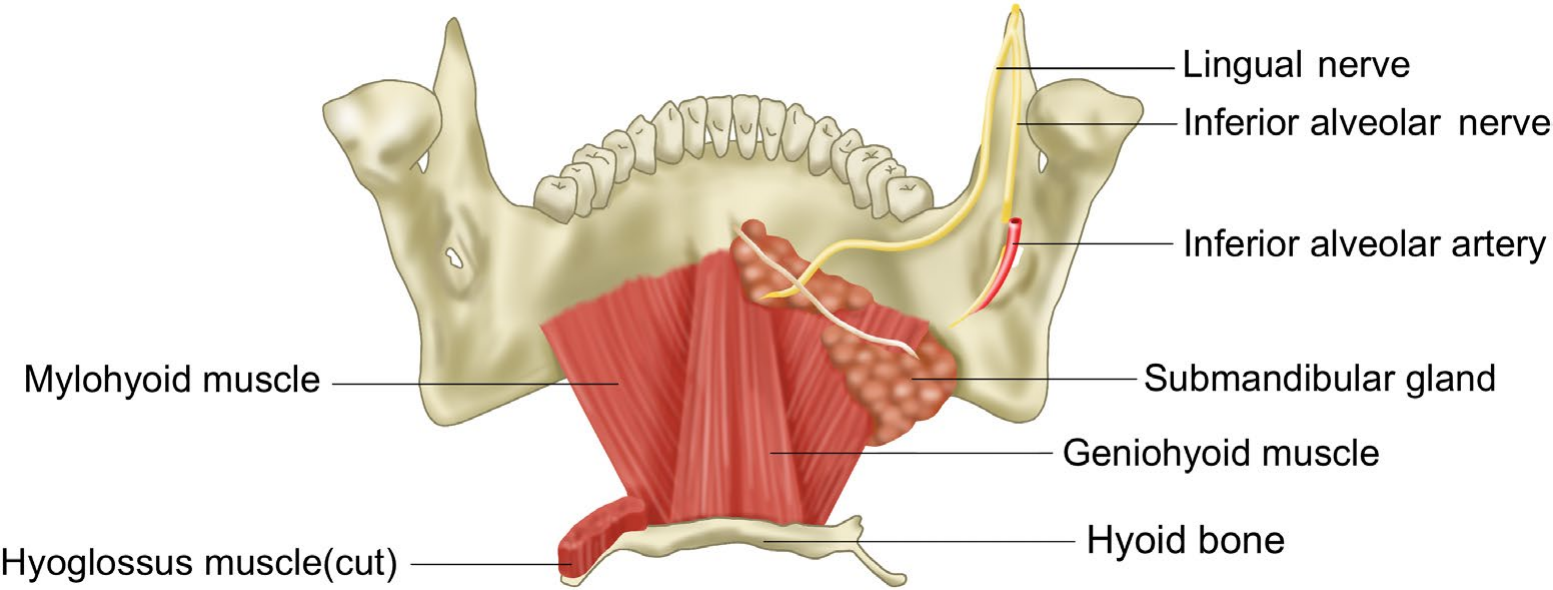
Schematic representation of the internal mandibular musculature, highlighting the relationships between the geniohyoid and digastric muscles in maintaining submental support.

Post‐surgical double chin deformity can occur following orthognathic surgery or genioplasty. Mandibular setback procedures can mimic congenital retrognathia, often with more pronounced skin laxity. Conversely, chin advancement surgeries may disrupt the muscular support of internal soft tissues, leading to soft tissue protrusion. Detachment of muscles inserting on the chin, such as the geniohyoid and anterior belly of the digastric, can exacerbate submental fullness (Figure 4).

The most common presentation of double chin deformity is associated with weight gain or localized cervical adiposity. This typically manifests as diffuse submental and cervical fullness, rather than isolated central submental protrusion.

These etiologies can occur in isolation or in combination. Clinical presentations often involve complex interactions, such as congenital muscular insufficiency exacerbated by weight gain, or post‐surgical skin laxity complicated by cervical fat accumulation. Given the multifactorial nature of double chin deformity, a thorough understanding of the underlying causes is crucial for tailoring appropriate treatment strategies.

**FIGURE 5:**
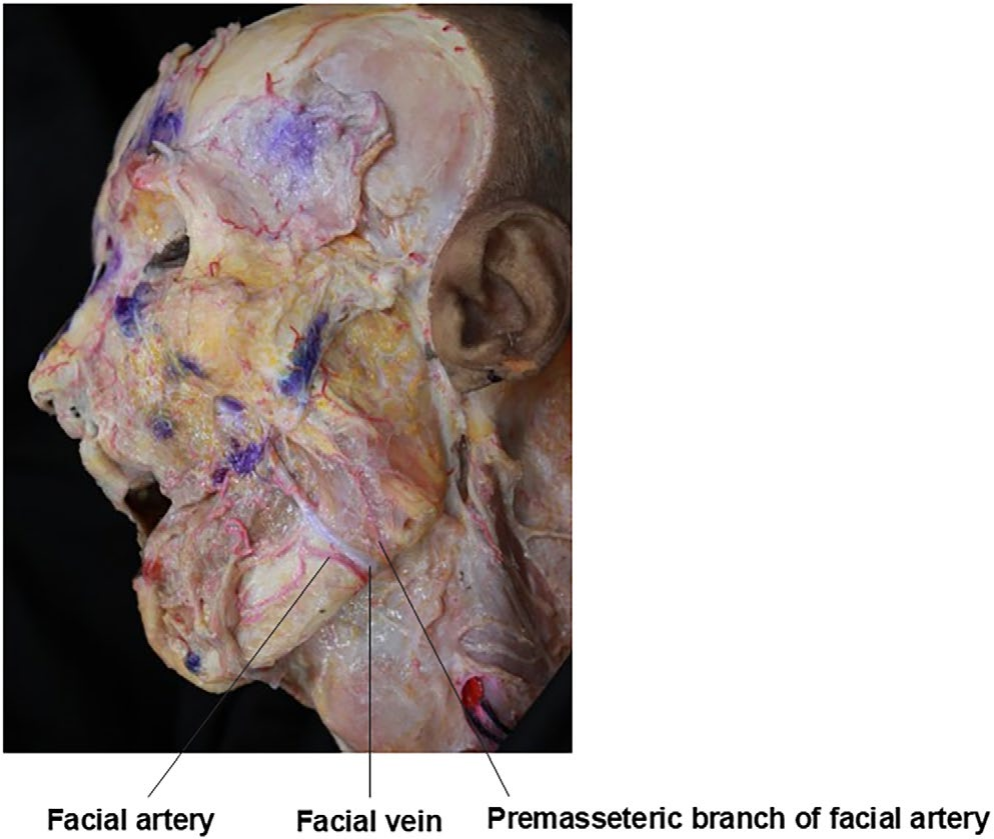
Vascular mapping showing the course and relationships of the facial artery, facial vein, and premasseteric branch of the facial artery in the submandibular region.

Regarding anatomical considerations for barbed thread lifting in double chin correction, attention must be given to the neurovascular structures traversing the mandibular‐cervical junction. The antegonial notch, a slight depression at the junction of the mandibular body and angle, can be palpated near the inferior border of the masseter muscle. During submental procedures, care must be taken to avoid injury to the facial artery and vein, as well as the premasseteric branch of the facial artery, which pass through this notch to supply the lateral lower face (Figure 5).

**FIGURE 6:**
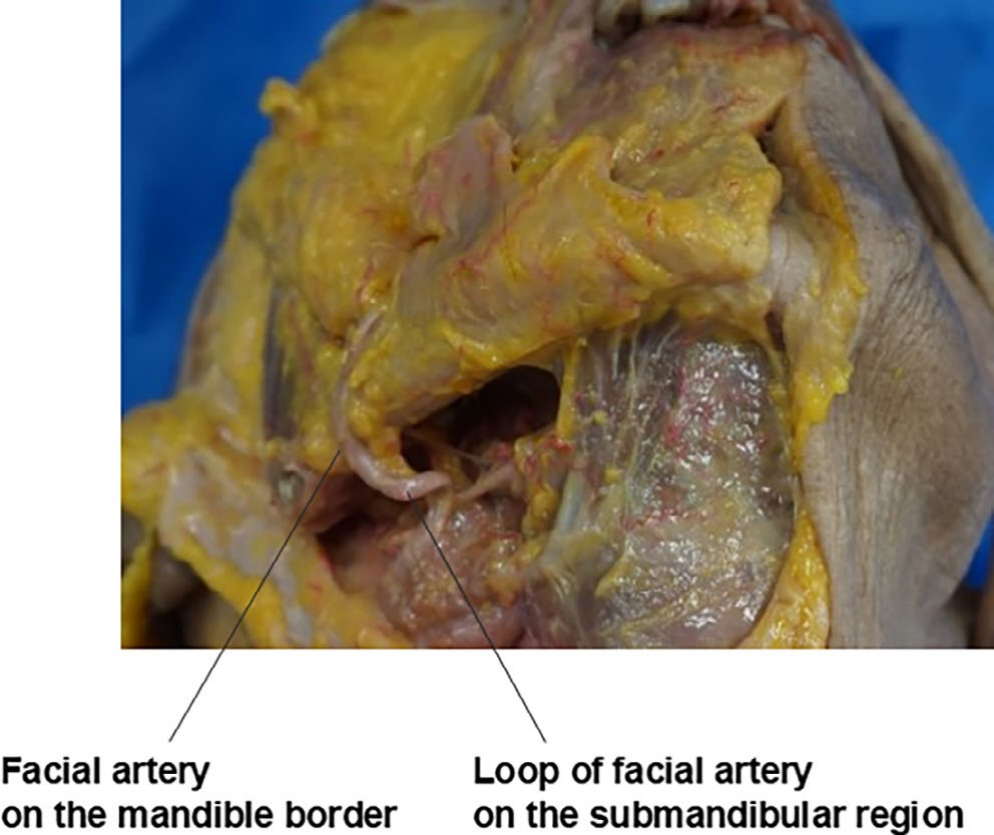
Detailed illustration of the facial artery's characteristic loop formation as it traverses the inferior border of the mandible, critical for safe thread passage.

The course of these vascular structures is as follows: The facial artery originates from the external carotid artery in the carotid triangle of the neck, just superior to the greater cornu of the hyoid bone. It forms a loop in the submandibular region before winding around the lower border of the mandible and traversing the antegonial notch to enter the face (Figure 6). As it ascends, the facial artery exhibits a tortuous course, weaving above and below the mimetic muscles and crossing the nasolabial fold medially and laterally while giving off various branches to the nose and lips.

**FIGURE 7:**
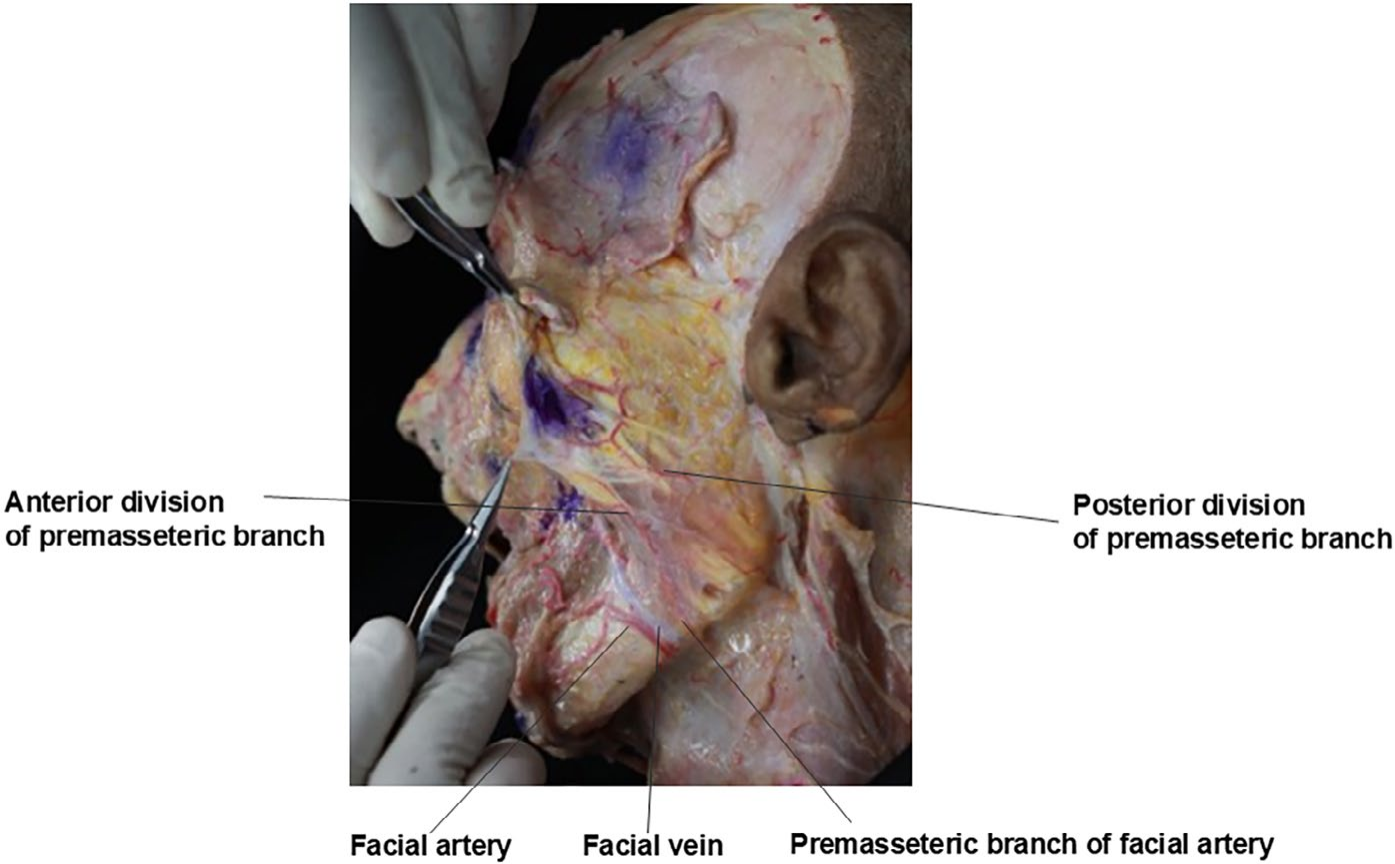
Anatomical diagram showing the branching pattern of the premasseteric artery into anterior and posterior divisions and their relationships to surrounding structures.

Before the facial artery turns medially after passing the antegonial notch, it gives rise to the premasseteric branch, which ascends obliquely towards the anterior border of the masseter muscle. The premasseteric branch typically originates approximately 2 cm superior to the mandibular base and 2 cm anterior to the masseter muscle. It bifurcates into anterior and posterior branches near its junction with the masseter. The anterior branch traverses the buccal fat pad between the zygomaticus major and masseter muscles, supplying the buccal region. The posterior branch follows a tortuous course posteriorly to supply the masseter muscle. Variations may include anastomoses with the infraorbital artery at the medial angle of the eye (Figure 7).

In contrast to the tortuous facial artery, the facial vein follows a relatively straight course, typically positioned about 1.5 cm posterior to the facial artery in the lower face (Figure 5).

During procedures involving the submental and mandibular border regions, caution must be exercised to avoid injury to these major vessels. Fortunately, as they traverse the mandible, these vessels lie deep to the platysma muscle, close to the bone. Therefore, thread lifting procedures focusing on the superficial fat layer above the platysma in the mandibular border region are relatively safe.

**FIGURE 8:**
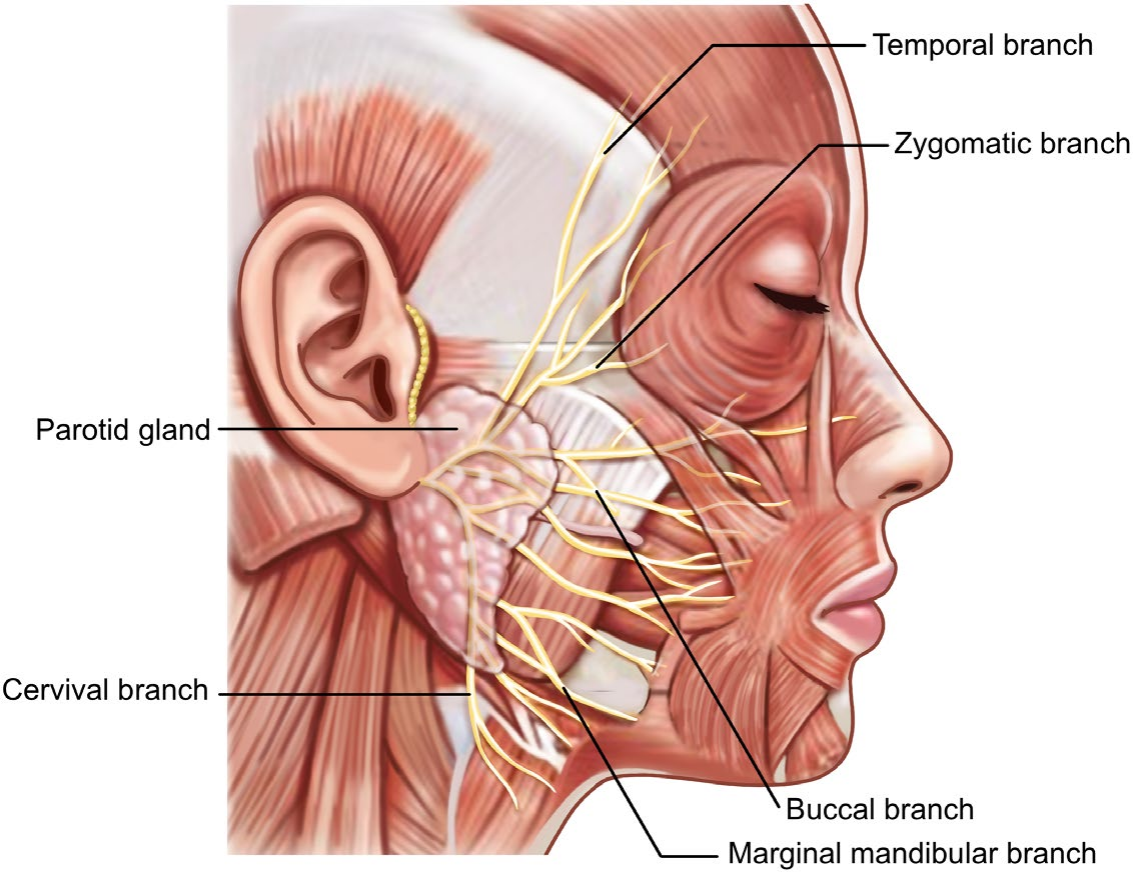
Branching pattern of the facial nerve within the parotid gland, illustrating its five major divisions and their courses through the facial tissues.

Regarding the nervous anatomy, the motor innervation to the mandibular border and submental regions is provided by the marginal mandibular (fourth) and cervical (fifth) branches of the facial nerve. After dividing into five branches within the parotid gland, the facial nerve typically emerges at the gland's edge, coursing deep to the SMAS and generally running along the deep aspect of the facial mimetic muscles (Figure 8). The marginal mandibular branch innervates the lower part of the orbicularis oris, depressor anguli oris, depressor labii inferioris, and mentalis muscles, while the cervical branch supplies the platysma muscle. Like the vasculature, these nerves course deep to the muscle layer. Therefore, maintaining procedures within the superficial fat layer significantly reduces the risk of neural injury.

Regarding sensory innervation, during double chin barbed thread lifting procedures where the thread's anchor point is set in the tough mastoid fascia covering the mastoid bone below the ear, the thread traverses the sternocleidomastoid (SCM) muscle posterior to the mandibular ramus. This trajectory poses a risk of injury to the great auricular nerve, which obliquely crosses the SCM muscle.

**FIGURE 9:**
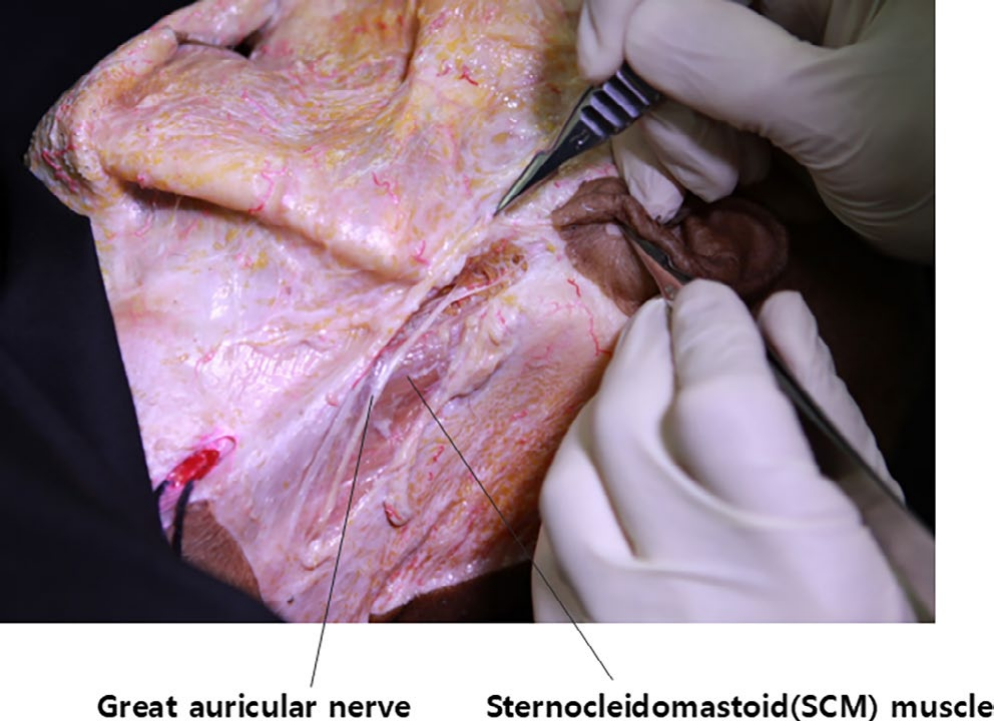
Topographical anatomy showing the precise location and course of the great auricular nerve across the sternocleidomastoid muscle.

The great auricular nerve, a sensory branch derived from the second and third cervical nerves, ascends over the anterior surface of the SCM muscle. It provides sensory innervation to the majority of the ear, excluding the region supplied by the auriculotemporal nerve (Figure 9).

**FIGURE 10:**
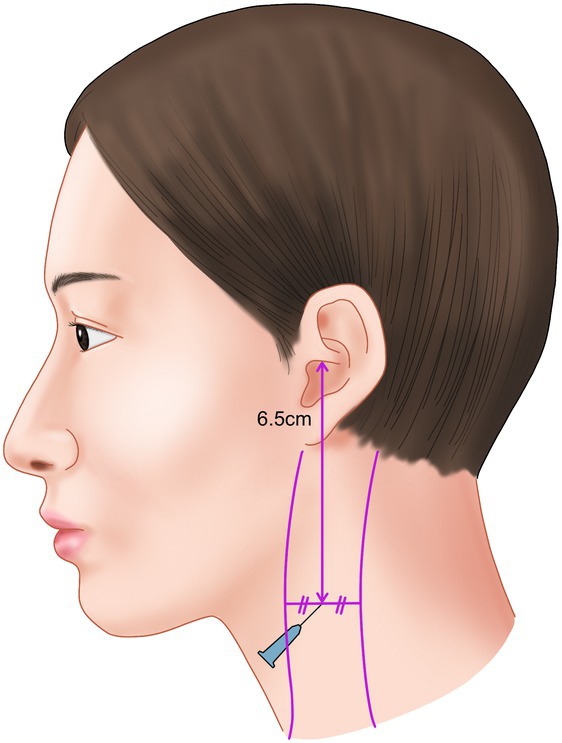
Technical illustration demonstrating the optimal injection point for great auricular nerve block, approximately 6.5 cm inferior to the external auditory canal.

To localize the nerve's course across the muscle, the patient should be positioned supine with the head rotated contralaterally to accentuate the SCM muscle. After marking the anterior and posterior borders of the muscle, the point where the nerve crosses can be approximated at the midpoint between these borders, approximately 6.5 cm inferior to the external auditory canal. This landmark is also utilized for great auricular nerve blocks (Figure 10).

When performing double chin barbed thread lifting using the traditional method of anchoring to the mastoid fascia, traversing the SCM muscle is inevitable. It is important to note that there is no absolutely foolproof method to ensure the complete safety of the great auricular nerve from needle or cannula injury during this procedure.

In double chin barbed thread lifting procedures, the anatomical structures requiring the utmost caution are actually the salivary glands—specifically, the parotid and submandibular glands—rather than nerves and blood vessels.

**FIGURE 11:**
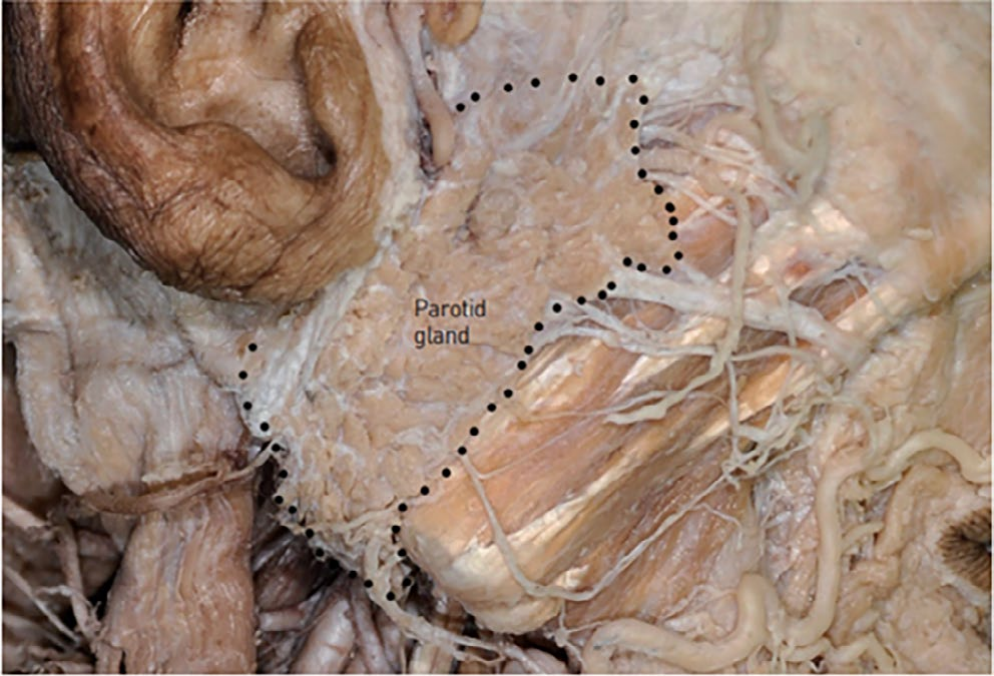
Anatomical boundaries and extent of the parotid gland, including its relationship to the zygomatic arch and mandibular angle.

**FIGURE 12:**
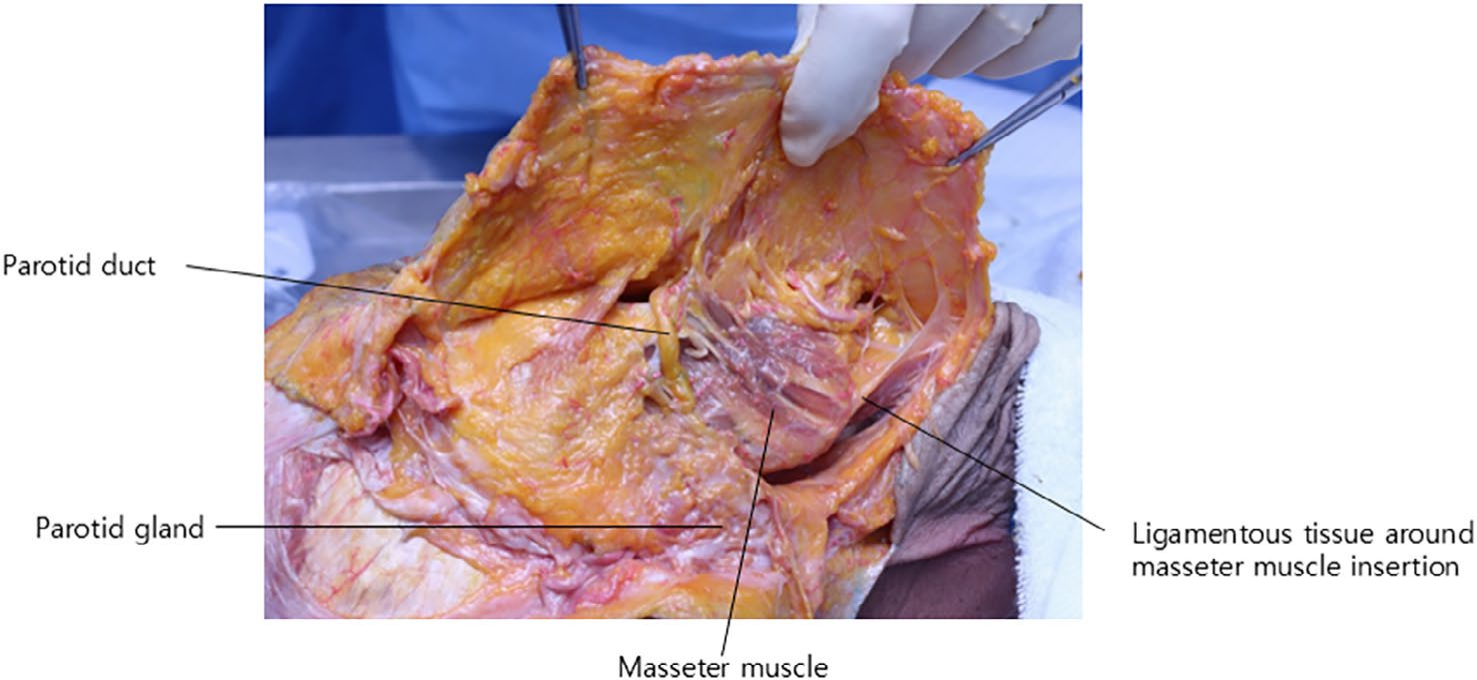
Cross‐sectional anatomy showing the depth relationships between the parotid gland and masseter muscle, crucial for safe thread placement.

The parotid gland is typically bounded superiorly by the zygomatic arch, posteriorly by the ear lobe, and inferiorly by the mandibular border. However, it occasionally extends beyond the posterior border of the mandibular ramus, creating a visible fullness below the ear (Figure 11). The parotid gland is situated deep to the parotid‐masseteric fascia, which lies beneath the facial SMAS layer. The glandular tissue is further encapsulated by the parotid capsule. During thread lifting procedures, it is crucial to avoid penetrating this capsule. When passing a thread posterior to the mandibular angle and beneath the ear, the needle or cannula should be advanced parallel to the skin surface and superficially to prevent parotid gland injury (Figure 12) [25, 26, 27].

**FIGURE 13:**
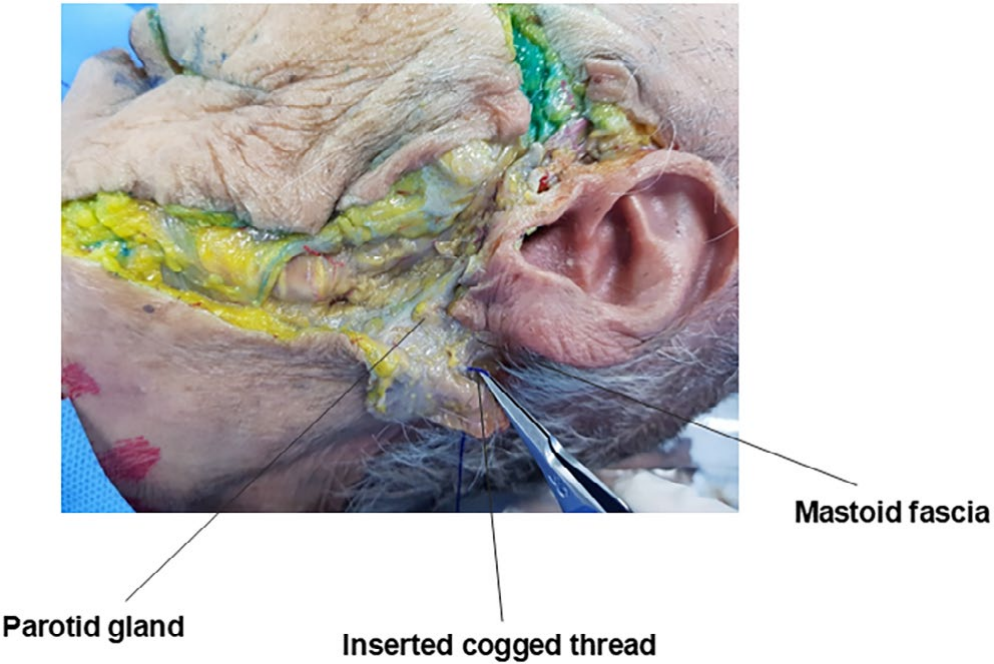
Technical diagram illustrating potential complications of thread insertion through the parotid gland during mastoid fascia anchoring in double chin lifting.

However, as previously mentioned, when using the mastoid fascia as the fixing point for double chin thread lifting, the needle or cannula inevitably needs to pass deeper than the subcutaneous fat layer beneath the ear, increasing the risk of parotid gland penetration. To safely traverse this area, the instrument must be kept as close to the skin as possible, which can make it challenging to secure the thread to the firm tissue beneath the ear (Figure 13).

**FIGURE 14:**
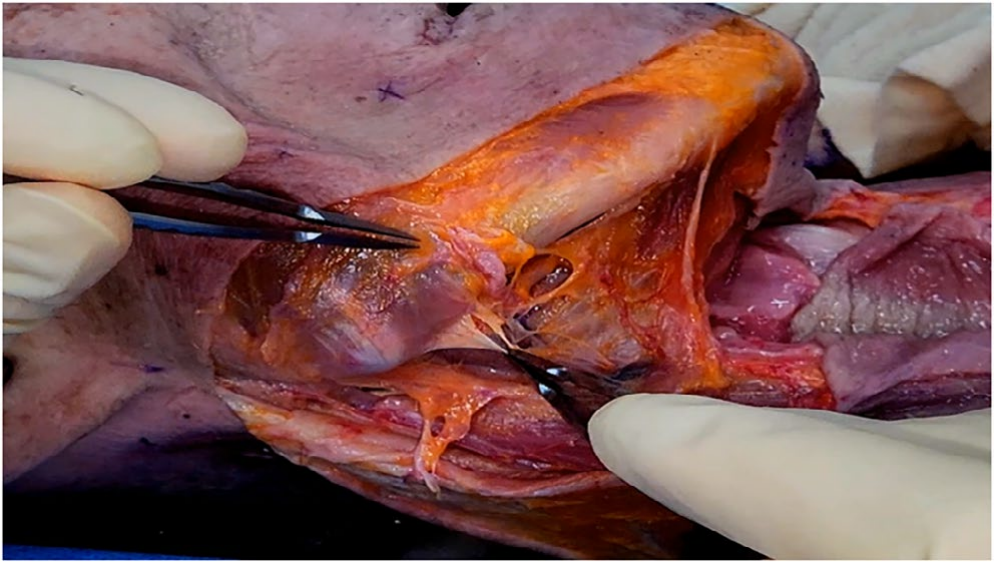
Anatomical illustration highlighting the subangular deep fascia as an optimal fixing point for double chin thread lifting, avoiding critical structures.

Consequently, the author now prefers to use the tough ligamentous tissue formed at the insertion of the masseter muscle (Figure 12) and the firm fascial tissue on the medial aspect of the mandible as fixation points for double chin thread lifting, rather than the mastoid fascia posterior‐inferior to the ear (Figure 14).

**FIGURE 15:**
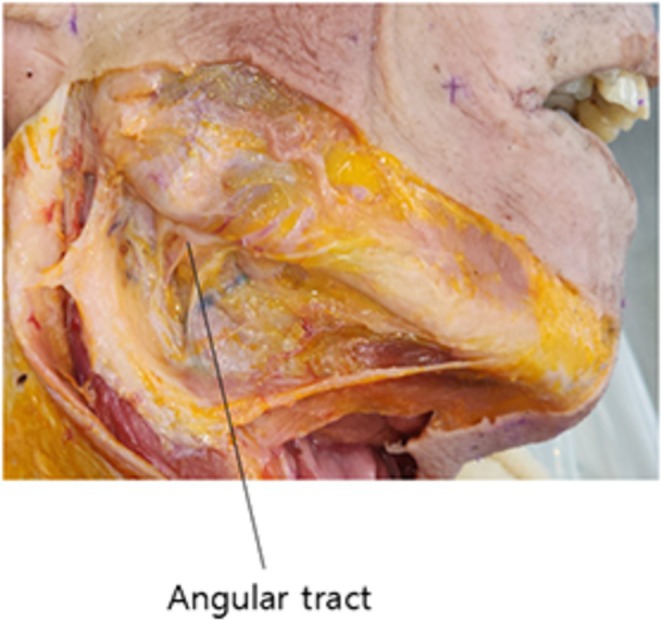
Detailed mapping of the angular tract in the submandibular region, showing its relationship to surrounding anatomical structures.

It's important to note that the subplatysmal region of the neck contains the cervical fascia, a deep fascia that protects the underlying deep muscles. Particularly, medial to the mandibular angle, between the submandibular gland and the lower portion of the parotid gland, there exists a strong fascial band continuous with the fascia attaching to the posterior belly of the digastric muscle. In otolaryngology, this structure is referred to as the angular tract (Figure 15).

The angular tract serves as a crucial landmark for protecting the submandibular gland during submandibular region surgeries. Medial to the mandibular angle, there exists a deep fascia continuous with this angular tract, which can be termed the subangular deep fascia. This robust tissue can serve as a novel fixation point for double chin barbed thread lifting. Deep palpation of the tissues medial to the mandibular angle, including the platysma muscle, reveals an immobile, firm, and fibrous tissue, which constitutes the subangular deep fascia (Figure 14).

Utilizing this subangular deep fascia as an anchor point for double chin thread lifting offers several advantages:

1. It eliminates the need for the thread to traverse the sternocleidomastoid muscle, thereby fundamentally avoiding potential injury to the great auricular nerve.
2. It is situated anterior to the parotid gland and slightly medial to the mandibular bone, rather than lateral where the parotid gland is located, thus minimizing the risk of parotid gland penetration.

**FIGURE 16:**
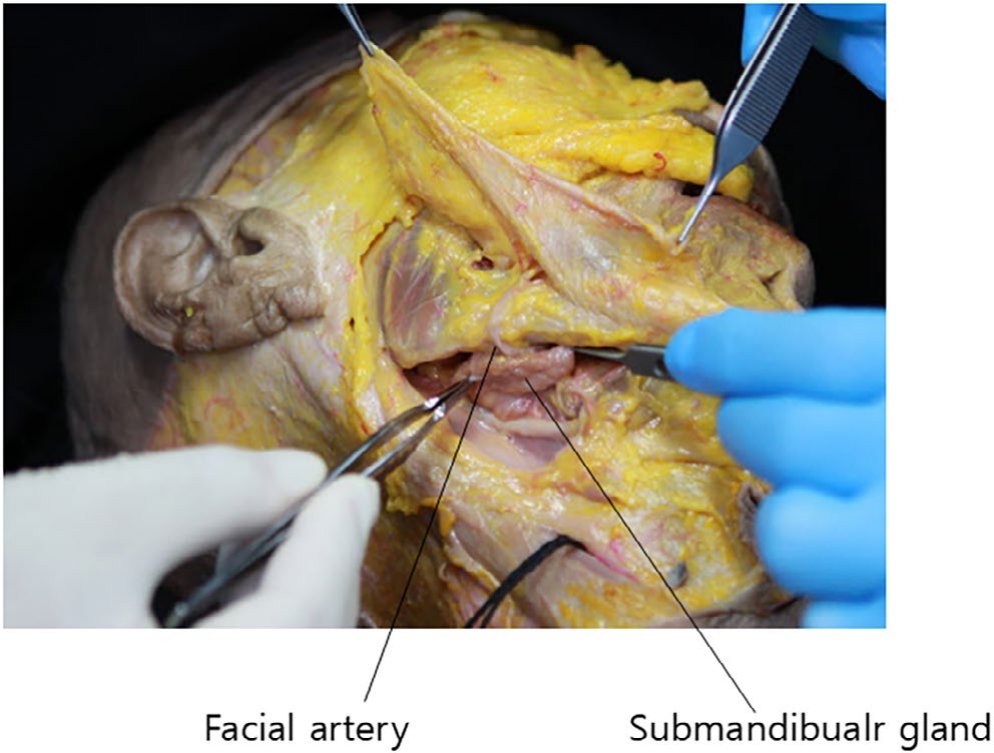
Anatomical diagram depicting the location and relationships of the submandibular gland, essential for differential diagnosis of submental fullness.

A critical differential diagnosis in double chin deformity is submandibular gland hypertrophy. This condition typically presents as a characteristic convexity in the posterior two‐thirds of the mandible, rather than in the central submental region (Figure 16). The submandibular gland is located deep to the posterior two‐thirds of the mandibular border. While not readily visible, it can be palpated as a slightly firm mass when the patient swallows.

During double chin correction procedures, care must be taken not to penetrate too deeply into the subplatysmal layer, which feels slightly softer than the overlying muscle. Adhering to this precaution significantly reduces the risk of inadvertently injuring the submandibular gland.

Submandibular gland hypertrophy is more noticeable in patients with less submental fat and thin skin. It is crucial not to misidentify this condition as a double chin deformity, as thread lifting would be ineffective and potentially harmful in such cases. For patients with submandibular gland hypertrophy, botulinum toxin injection is a more appropriate treatment. Typically, 10–15 units of botulinum toxin per side, administered via a 29G 0.5‐in. needle at 4–5 points around the prominent area, can effectively reduce the fullness.

## 3 Procedural Design and Technique

Prior to performing barbed thread lifting for double chin correction, it is advisable to reduce excessive fat volume through liposuction, fat‐dissolving injections, or energy‐based devices for fat reduction. This is crucial because, despite the ability of thread lifting to reposition central submental fat and soft tissue laterally or towards the ears, an excessive absolute fat volume may lead to recurrence of fullness as the lifting effect diminishes over time.

Pre‐procedural assessment can be significantly enhanced through dermatologic ultrasound, which has emerged as a valuable tool for thread lifting procedures. As described in previous studies, ultrasound enables precise evaluation of pre‐platysmal and post‐platysmal fat distribution, accurate identification of platysmal decussation patterns (Types I, II, and III), and detection of anatomical variations such as ectopic parotid or submandibular gland tissue. For optimal visualization, a high‐frequency linear transducer (15–22 MHz) is recommended, providing excellent resolution of superficial facial layers [27, 28]. Ultrasound imaging allows for mapping of critical neurovascular structures, particularly at the antegonial notch where the facial artery and vein traverse [28]. This detailed anatomical mapping may substantially reduce the risk of complications [18]. For patients with previous interventions, ultrasound can identify areas of fibrosis or adhesion between tissue planes, allowing for strategic modification of thread placement techniques.

**FIGURE 17:**
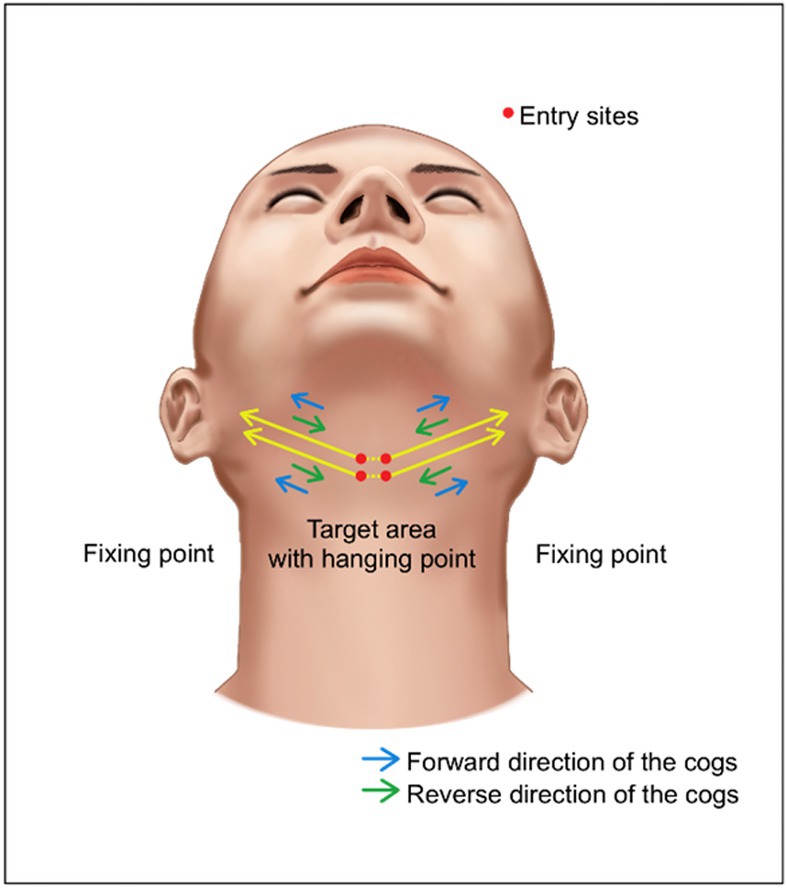
Technical illustration showing thread placement design for double chin lifting using reverse technique with I‐shaped cogged threads. (The tread that has been used is LVDR LIFT, Sihler Inc., Seoul, Korea and The thread that is used are 10 cm length of Secret Illusion (Hyundae Meditech Inc., Wonju, Republic of Korea) and LVDR, Sihler thread (Sihler Inc., Seoul, Republic of Korea).

The authors currently employ two primary techniques for double chin barbed thread lifting, depending on the type of thread used:

The first method utilizes 10 cm I‐shaped bidirectional cogged threads. An entry site is created at the midpoint of the neck, through which two threads are inserted in a reverse manner, extending from the center laterally on both sides. To maximize traction on the central fatty tissue, two central entry points are created, as illustrated, allowing the medial aspects of the left and right threads to overlap and create a synergistic lifting effect on the central fullness (Figure 17). As the threads are inserted from medial‐to‐lateral, rather than lateral to medial, reverse technique‐specific I‐shaped threads with a higher cog density on the handle side are used. This design ensures greater engagement with the lax medial tissues, providing superior efficacy compared to traditional bidirectional cogged threads with uniform cog distribution.

**FIGURE 18:**
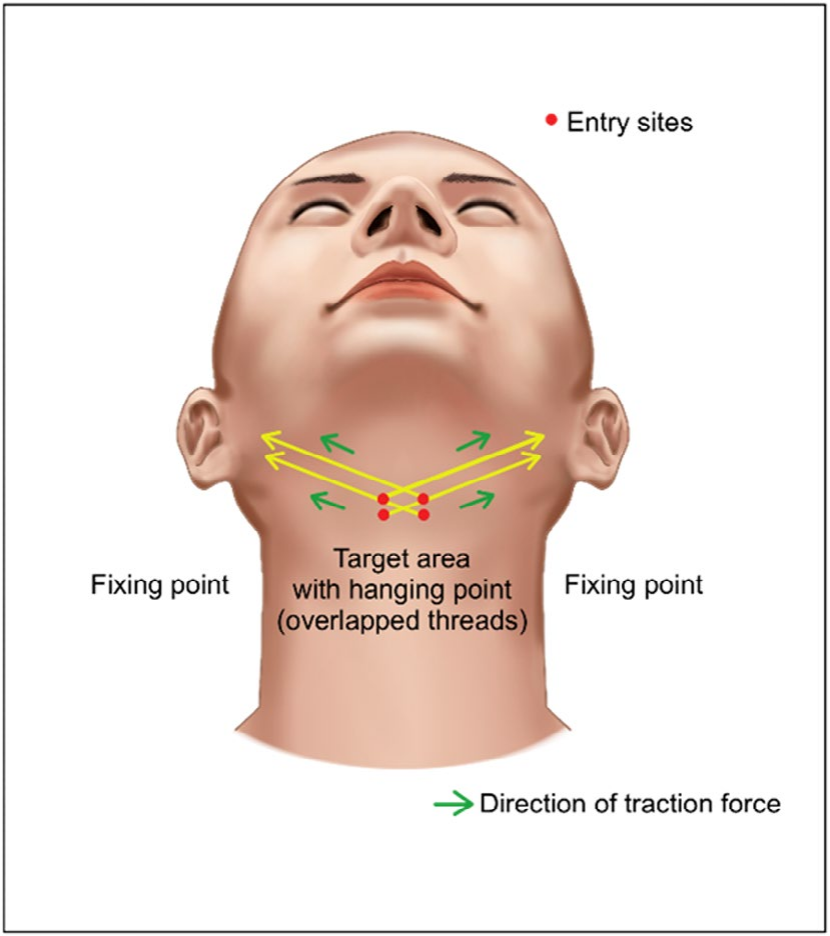
Procedural design diagram for double chin lifting using double arm needle U‐shaped long bidirectional cogged thread placement. The thread that is used are double arm thread (Hyundae Meditech Inc., Wonju, Republic of Korea).

The second technique employs a long U‐shaped double arm needle bidirectional cogged thread. The thread is straightened, and a central entry site is created in the midneck region. The cog‐free central portion of the thread is positioned at the neck midline, and the bilateral cogged portions are then extended laterally from the central submental region (Figure 18). While the author previously utilized a guide cannula for separate thread insertion, with increased procedural familiarity, the U‐shaped long thread with bilateral needles is now preferred for its efficiency and simplicity.

When creating two central entry points rather than one for the insertion of the central thread, it is advantageous to select a double arm needle U‐shaped long cogged thread with an appropriate length of cog‐free central portion. This allows the cog‐free segment to be positioned between the two entry points, maximizing compression of the central fatty tissue.

The author employs both techniques, inserting the threads from the central submental region and extending them laterally. While this approach necessitates creating incisions in the central area of the double chin, potentially resulting in more noticeable entry points initially and a slightly more complex procedure, it offers long‐term benefits. The dimples that form around the central entry points in the early post‐procedural period serve to compress and anchor the central fullness medially. From a long‐term perspective, this contributes positively to the improvement of the double chin deformity.

The procedure is performed with the patient initially seated to mark the area of most significant double chin deformity. The patient is then reclined with a thin pillow under the shoulders to slightly elevate the chin. For creating entry points, the author prefers using a small needle for initial puncture, followed by cannula dilation, rather than using a large needle or awl. This step is crucial to prevent severe dimpling caused by skin engagement with the thread cogs.

The technique using short I‐shaped cogged threads, while simpler due to the use of cannula‐mounted threads, may have less efficacy and longevity compared to U‐shaped threads as the lateral fixation point is not penetrated. Typically, four to eight reverse‐specific I‐shaped bidirectional cogged threads are used, with 1–2 threads inserted in an alternating pattern at each of the four entry points (two superior and two inferior) in the central submental region.

The lateral fixing point for inserted threads should be the subangular deep fascia medial to the mandibular angle, with the cannula tip penetrating the firm tissue beneath the platysma muscle. The subangular deep fascia, while clearly identifiable in anatomical studies, requires specific approaches for reliable access in clinical practice. Practitioners can consistently locate this anchor point through firm palpation approximately 1–1.5 cm medial to the mandibular angle, just beneath the mandibular border. The cannula can be initially inserted at a 90° angle to reach this subangular deep fascia, then advanced at approximately 20°. Ultrasound guidance may be helpful in cases of suspected anatomical variations, such as ectopic parotid tissue. The cannula should traverse above the platysma muscle between the entry and fixation points to avoid critical neurovascular structures while engaging the deep portion of the fat tissue for maximum traction. After thread insertion, the excess thread at the entry point is gently pulled and trimmed to prevent protrusion as swelling subsides. Taping may be applied as needed based on the degree of submental fullness and post‐procedural edema.

For the U‐shaped long cogged thread with bidirectional needles, the creation of four entry points (two superior and two inferior) in the central submental region is similar to the I‐shaped thread technique. The central portion of the extended thread is positioned subcutaneously, with the lateral ends exteriorized through the fixation points and trimmed. The degree of thread traction and trimming should be determined with the patient in an upright position to ensure symmetry and appropriate tension. Excessive tension can cause discomfort during deglutition and daily activities, while insufficient tension may compromise efficacy. When trimming the exteriorized thread ends laterally, minimal traction should be applied to reduce the risk of thread protrusion. The bilateral symmetry of this extended thread technique reduces the likelihood of thread migration compared to individually placed short I‐shaped threads.

Novice practitioners may find it challenging to sense the long needle traversing along the platysma muscle when using U‐shaped long cogged threads with bidirectional needles. Moreover, once the thread is inserted, it cannot be withdrawn. Therefore, until sufficient experience is gained, using I‐shaped short cogged threads mounted on cannulas may be safer. The blunt cannula tip facilitates accurate layer placement, reduces bleeding, and allows for repositioning if the initial placement is suboptimal.

Post‐procedural discomfort, including a sensation of neck constriction and difficulty with certain neck movements or wide mouth opening, typically persists for 1–3 days. The technique using two short I‐shaped threads crossing in the center generally causes less central constriction than the long thread method, which can produce a more pronounced strangling sensation if overtightened. Patients with adhesions between the platysma muscle and skin from previous procedures, such as liposuction, may experience more severe discomfort. As the long U‐shaped thread technique may result in more pronounced symptoms, a 3‐day course of anti‐inflammatory analgesics is recommended post‐procedure.

Central dimpling around the entry points typically resolves spontaneously within 1–2 weeks, unless caused by direct skin engagement with thread cogs. Most discomfort subsides after 1 week post‐procedure. The duration of the aesthetic effect varies, but patients with moderate volume and thin skin may maintain results for over 6 months. However, in patients with excessive fat and thick skin, effects may diminish around 2 months post‐procedure. Therefore, pre‐procedural assessment of submental fat volume and skin laxity, coupled with thorough patient counseling regarding expected outcomes, is crucial.

The efficacy and longevity of the procedure can vary significantly between patients. Those with moderate volume and thin skin may maintain results for over 6 months, while patients with excessive fat and thick skin might see effects diminish around 2 months post‐procedure. Therefore, a thorough pre‐procedural assessment of submental fat volume and skin laxity, coupled with comprehensive patient counseling regarding expected outcomes, is essential for managing expectations and ensuring patient satisfaction.

## 4 Discussion

The anatomical complexity of the submental region necessitates a precise understanding of neurovascular relationships and fascial planes for safe and effective thread placement. The identification and utilization of the subangular deep fascia as an anchor point represent a significant advancement in technique safety, particularly in avoiding the great auricular nerve and parotid gland [25, 26, 27].

Pre‐procedural volume assessment and reduction through complementary treatments such as liposuction or fat‐dissolving injections play crucial roles in optimizing outcomes. This approach addresses the fundamental volume component of double chin deformity before attempting structural repositioning through thread lifting.

The integration of dermatologic ultrasound into pre‐ and post‐procedural assessment represents an important advancement in both the safety and efficacy of double chin thread lifting. By providing detailed visualization of the relationship between fascia, muscle, fat compartments, and neurovascular structures, ultrasound guidance may help practitioners achieve more precise thread placement while minimizing the risk of complications. Post‐procedural ultrasound may be helpful in identifying complications such as misplaced threads, parotid injury, or fibrosis. Particularly for procedures targeting the subangular deep fascia, ultrasound enables confirmation of proper needle/cannula positioning relative to the parotid gland and submandibular structures [18, 27].

The choice between I‐shaped and U‐shaped thread techniques significantly impacts both procedural complexity and outcomes. While I‐shaped threads offer greater safety for novice practitioners, U‐shaped threads may provide superior long‐term results when properly placed [29, 30].

Post‐procedural management and patient education regarding temporary symptoms and expected recovery timeline are essential components of successful treatment. Understanding the varying duration of aesthetic effects based on individual patient characteristics helps manage expectations [28, 31].

The integration of anatomical knowledge with technical expertise represents the cornerstone of successful double chin thread lifting. Proper patient selection, considering factors such as skin thickness, fat volume, and platysmal anatomy, remains crucial for achieving optimal and lasting results.

## Author Contributions

**Gi‐Woong Hong, Kyu‐Ho Yi, Jovian Wan, and Song‐Eun Yoon:** conceptualization. **Gi‐Woong Hong, Jovian Wan, and Sky Wong:** writing – original draft preparation. **Gi‐Woong Hong, Kyu‐Ho Yi, and Song‐Eun Yoon:** writing – review and editing. **Gi‐Woong Hong and Kyu‐Ho Yi:** visualization. **Gi‐Woong Hong and Kyu‐Ho Yi:** supervision. All authors have reviewed and approved the article for submission.

## Conflicts of Interest

The authors declare no conflicts of interest.

## Data Availability

The data that support the findings of this study are available on request from the corresponding author. The data are not publicly available due to privacy or ethical restrictions.
